# Genomic Selective Constraints in Murid Noncoding DNA

**DOI:** 10.1371/journal.pgen.0020204

**Published:** 2006-11-24

**Authors:** Daniel J Gaffney, Peter D Keightley

**Affiliations:** 1 Institute of Evolutionary Biology, Ashworth Laboratories, School of Biological Sciences, University of Edinburgh, Edinburgh, United Kingdom; 2 McGill University and Genome Quebec Innovation Centre, Montréal, Québec, Canada; University of Oxford, United Kingdom

## Abstract

Recent work has suggested that there are many more selectively constrained, functional noncoding than coding sites in mammalian genomes. However, little is known about how selective constraint varies amongst different classes of noncoding DNA. We estimated the magnitude of selective constraint on a large dataset of mouse-rat gene orthologs and their surrounding noncoding DNA. Our analysis indicates that there are more than three times as many selectively constrained, nonrepetitive sites within noncoding DNA as in coding DNA in murids. The majority of these constrained noncoding sites appear to be located within intergenic regions, at distances greater than 5 kilobases from known genes. Our study also shows that in murids, intron length and mean intronic selective constraint are negatively correlated with intron ordinal number. Our results therefore suggest that functional intronic sites tend to accumulate toward the 5′ end of murid genes. Our analysis also reveals that mean number of selectively constrained noncoding sites varies substantially with the function of the adjacent gene. We find that, among others, developmental and neuronal genes are associated with the greatest numbers of putatively functional noncoding sites compared with genes involved in electron transport and a variety of metabolic processes. Combining our estimates of the total number of constrained coding and noncoding bases we calculate that over twice as many deleterious mutations have occurred in intergenic regions as in known genic sequence and that the total genomic deleterious point mutation rate is 0.91 per diploid genome, per generation. This estimated rate is over twice as large as a previous estimate in murids.

## Synopsis

Most DNA can typically be divided into two categories: regions that encode the instructions for the assembly of a protein molecule (protein-coding genes) and those that do not (noncoding). Although mammalian genomes are primarily noncoding, relatively little is known about how much of this is functional, where such regions are found in the genome, and what functions they are likely to perform. In this study, the authors investigated the quantity and location of functional noncoding DNA in mice and rats. They estimate that functional noncoding DNA is at least three times as common as coding DNA in rodents, and the majority is located large distances from known protein-coding genes. Putatively functional intronic DNA tends to be clustered towards the gene 5′ end, suggesting that much intronic sequence is instrumental in regulating gene expression. This study also finds that genes involved in development and the nervous system are typically associated with much higher quantities of functional noncoding DNA, suggesting that these genes require more finely tuned control of their expression. One implication of this study is the finding that disease-causing mutations have occurred more frequently in noncoding regions and may have affected gene expression, rather than protein structure.

## Introduction

Protein-coding genes typically comprise a rather small part of many mammalian genomes [1–4]. Although it has been known for some time that at least some of the noncoding portion of the genome must be functional, the significance and nature of the encoded function has remained elusive.

Large-scale comparative genomic studies are, however, beginning to reveal the extent of potentially functional noncoding DNA. The publication of the draft mouse genome sequence enabled the first genome-wide comparative analysis of mammalian (human-mouse) noncoding DNA [2]. One intriguing suggestion arising from this study is that, although approximately 5% of small (50-base pair [bp]) segments in the human genome are under purifying selection, less than half of these segments are located in protein-coding sequence.

Much work has since been undertaken in an attempt to elucidate the nature of this functional noncoding DNA. In particular, large-scale computational comparative analyses have revealed extensive evolutionary conservation of noncoding DNA in multiple mammalian and other species [5–13]. In addition to computational approaches, high-density oligonucleotide arrays have also revealed extensive conservation of nonrepetitive human sequences in other mammals [14]. Subsequent work has indicated that, whilst a number of conserved regions in noncoding DNA sequences may be undiscovered protein-coding genes, or partially overlap with existing genes [9], the evidence does not support a protein-coding function for such conserved regions in many cases [6,11]. It should be noted, however, that sequence conservation per se does not necessarily imply functionality and may reflect variation in the mutation rate [15].

One means of accounting for the effects of varying mutation rate involves calibration of the nucleotide substitution rate in putatively functional regions with that in a nearby sequence which is assumed to be evolving neutrally. This approach enables estimation of selective constraint, defined as the proportion of new mutations occurring at a locus which is strongly deleterious and removed by purifying selection [16], and of the rate of deleterious mutation, an important parameter in population genetics. This method has been previously employed, both to estimate the extent of purifying selection [2] and the genomic deleterious mutation rate in mammalian coding and noncoding DNA [17,18].

We adopted this approach to address the following questions: (i) How does the magnitude of selective constraint in murids vary between different types of noncoding DNA (e.g., introns, UTRs, and intergenic regions), (ii) What are the relative genomic contributions of the different classes of noncoding DNA to the total number of constrained bases in the murid genome, (iii) Do the numbers of selectively constrained noncoding sites vary with gene function, and (iv)What is the total genomic deleterious mutation rate per generation, *U,* in murids accounting for noncoding sites located large distances from known genic regions? We focused upon mice and rats because the genomic resources (annotation and sequence quality) for both mouse and rat are unparalleled by any other mammalian species pair. Furthermore, mouse and rat are sufficiently diverged, so that, whilst alignment of noncoding regions is not overly problematic, substantial statistical power is available for comparative genomics, unlike closely related species, such as human and chimpanzee.

We compiled a large dataset of mouse-rat gene orthologs and their surrounding noncoding DNA. Using a pair-wise comparison, we can, by definition, only infer the total number of selectively constrained bases in that portion of the mouse and rat genomes that can be aligned. However, mouse and rat are relatively closely related and the proportions of their genomes that can be aligned with one another represent the majority of both genomes [3]. Some previous studies of selective constraint have used a “fastest evolving” criterion to denote an assumed “neutral standard” [18–20]. This criterion assumes that those sites which appear to be evolving most swiftly are likely to be neutral, given that adaptive substitutions are likely to be negligibly rare. The validity of this assumption is crucial to the accurate estimation of selective constraint. In order to relax the “fastest-evolving” criterion of selective neutrality, observed nucleotide substitution rates in repetitive DNA were used throughout this study as an assumed neutral standard. In mammals at least, transposable elements (TEs) are likely to be highly enriched for neutrally evolving sequence.

One problem with using a “neutral standard” is that there is considerable compositional variation between TE families and different, nonrepetitive coding and noncoding DNA. Because the rate at which any one nucleotide mutates into another is known to vary between nucleotides [21,22], rates of nucleotide substitution may vary between neutrally evolving sequences whose base compositions differ. In this study, we attempted to determine to what extent varying base composition could explain differences in substitution rate observed under the null hypothesis of no selective constraints. To quantify this effect, we simulated the evolution of coding and noncoding sequences with realistic base compositions entirely free of selective constraints under a mutation model derived from mouse polymorphism data. Any differences in substitution we observed would therefore reflect the action of mutation coupled with compositional variation, as opposed to purifying selection.

## Results

The initial list of “known” ENSEMBL mouse genes contained 24,560 peptides. Processing to remove those peptides that did not meet our selection criteria left 8,932 mouse genes. Upon comparison with the rat genome, those matches that appeared to be invalid or matched a rat sequence that was not also a valid coding sequence (i.e., had one or more premature stop codons) were excluded. This left a total of 6,381 putative mouse-rat orthologous loci. Excluding masked bases (repetitive and putatively non-homologous), a total of 1.26 million 4-fold degenerate sites, 5.08-megabase (Mb) aligned sites from UTRs, 85.85-Mb aligned intronic sites, and 133.73 Mb of aligned intergenic sequence (of which 71.93 Mb was 5′ and 61.80 Mb 3′ intergenic) was extracted. The alignments also provided a total of 62.50 Mb of ancestral repeat sequence, of which 20.14 Mb was located within introns and the remaining 42.36 Mb located within intergenic regions. The total aligned sequence was 288.42 Mb, which is approximately 17% of the total alignable sequence between mouse and rat.

### Substitution Rates in Coding and Noncoding DNA

**Figure 1:**
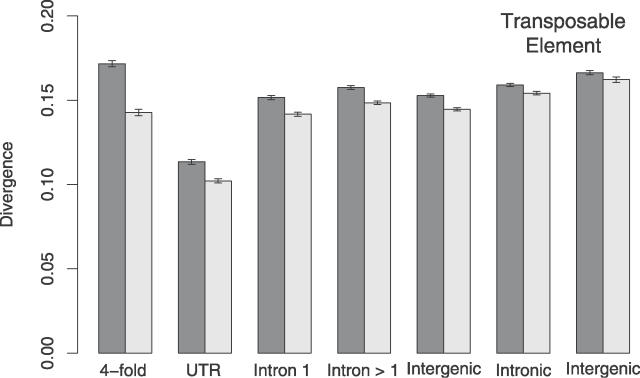
Mean Nucleotide Substitution Rates in Different Sequence Types Substitution rates were estimated at all sites (dark bars) and non-CpG-prone sites (light bars). Intronic substitution rates were estimated from all intronic sites, excluding splice regions which were assumed to occur in the first 20 and last 40 bp. 95% confidence intervals were estimated by bootstrapping the dataset by 1-Mb block, 1,000 times.

The mean nucleotide substitution rate was estimated for each of seven sequence classes: 4-fold degenerate sites, UTRs, first introns, non-first introns, intergenic DNA, and intronic and intergenic ancestral repeats. The results of this analysis are presented in Figure 1, which shows nucleotide substitution rates estimated at all sites and at non-CpG-prone sites. It is clear that the non-CpG-prone substitution rate is substantially lower than the substitution rate at all sites in all sequence classes. The widest margin between the two is observed at 4-fold degenerate sites and the smallest within intergenic repetitive DNA. This gradient reflects the variation in the CpG content of each sequence class. At non-CpG-prone sites, the most swiftly evolving sequence class is the ancestral repeats. This supports the assumption that after excluding CpG dinucleotides, TEs contain the highest proportion of neutrally evolving sites. The results also show a significantly higher (5% at non-CpG-prone sites) nucleotide substitution rate in those TEs located within intergenic DNA. One explanation of this result is a lower base mutation rate in transcribed DNA, possibly due to transcription-coupled repair of genes expressed in the germline [23].

### Simulation Results

**Figure 2:**
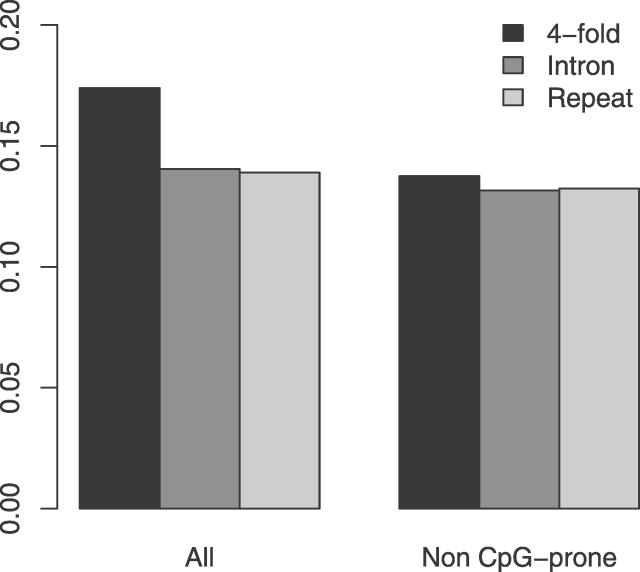
Substitution Rates at Simulated 4-Fold Degenerate, Intronic, and Repetitive Sites Means over 100 simulated replicates, each of which evolved a single sequence containing **~**8 Mb of coding, intronic, and repetitive sequence along two lineages are shown.

In order to investigate the effects of base composition and site selection on estimates of nucleotide substitution rates, 4-fold sites, introns, and ancestral repeats derived from real sequence data were simulated to evolve down two lineages entirely free of selective constraints. Mean nucleotide substitution rates, in each of the simulated sequence classes across 100 simulated phylogenies, are presented in Figure 2. At all sites, substitution rates are most substantially affected by differential frequencies of the CpG dinucleotide and are, therefore, highest at 4-fold degenerate sites, which have the highest CpG frequency (0.044 compared with 0.010 and 0.008 in introns and ancestral repeats, respectively). In addition to CpG hypermutation, our mutation model suggests that in murids, G/C bases are marginally more mutable than A/T bases (~1.36-fold). It is this that produces the decreasing gradient in non-CpG-prone substitution rates from 4-fold degenerate sites (% GC = 57.4), ancestral repeats (% GC = 44.5) to introns (% GC = 43.0).

Compared with the rates of nucleotide substitution observed in the real sequence data, a number of patterns are evident. Firstly, although 4-fold sites are clearly the most swiftly evolving class in the neutral simulations, this is not the case at non-CpG-prone sites in the real data (Figure 1). Whilst simulated 4-fold sites evolved ~7% faster than repetitive DNA, real 4-fold sites are in fact evolving ~7% slower than real intronic TEs. Thus, we may underestimate constraint at 4-fold sites, given that their base mutation rate, even excluding hypermutable CpG dinucleotides, may be somewhat higher than other regions of the genome.

Secondly, in the simulated phylogenies, TEs evolved marginally faster (~1.3%) than introns. This is also the case in the real data and suggests that at least some of the elevation of evolutionary rate observed in repetitive sequence over intronic DNA is due to neutral, mutational effects coupled with compositional variation. In order to quantify the potential effect of this compositional elevation of rates, we estimated “constraint” in simulated intronic sequence. For each simulated replicate, “constraint” was estimated in an identical fashion to the real data. Simulated TEs were used as a neutral standard to calculate the expected numbers of substitutions in the simulated intronic sequences, and this was compared to the observed rate. The distribution of estimated “constraint” across 100 replicates (Figure S1) suggests that positive constraint values in our real data, up to a maximum of ~1.4%, could be explained by mutation/compositional bias alone. Although the smallest difference observed between repetitive and nonrepetitive evolutionary rates (~3.8%; between intronic TEs and non-first introns, excluding splice regions) is still larger than that observed in simulated data, this result suggests caution in the interpretation of differences in substitution rate between sequences of even marginally different base composition.

### Variation in Ancestral Repeat Substitution Rates

**Figure 3:**
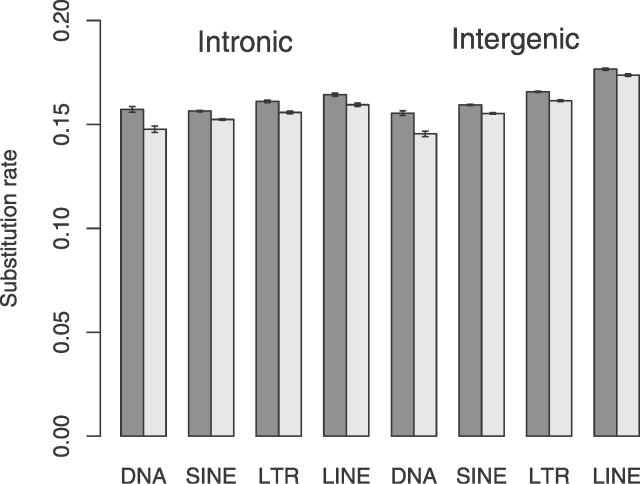
Estimated Mean Nucleotide Substitution Rate in Transposable Elements Substitution rates were estimated at all sites (dark bars) and non-CpG-prone sites (light bars). Elements are subdivided into those found in intronic and intergenic sequence. 95% confidence intervals were estimated by bootstrapping the dataset by 1-Mb block, 1,000 times.

We also investigated the validity of the assumption of neutral evolution in ancestral repetitive DNA. As part of this, the mean nucleotide substitution rate was estimated separately in each of the four main TE families (Figure 3). Under the assumption of selective neutrality and the same base mutation rate, the nucleotide substitution rates in each element class should be approximately the same. It is evident, however, that this is not the case, and there is significant variation in the mean element family substitution rates. This pattern is evident in both intronic and intergenic elements, although intergenic elements generally tend to be slightly more swiftly evolving than their intronic counterparts.

In order to determine whether the observed variation in nucleotide substitution between different TE families could be due to compositional variation coupled with mutational bias, we simulated the evolution of the four main element families under our mutation model. The results of these simulations suggest that the mutation model implemented in our simulations could not produce the substitutional variation we observe between real element families (Figure S2). In our real data the mean TE family substitution rate was lowest in DNA elements, followed by short INterspersed elements (SINEs) and long terminal repeat transposons (LTRs), and highest in long INterspersesd element (LINE) TEs. This is the case at all sites and non-CpG-prone sites. In contrast, in our simulated TE families, at all sites we observed the highest estimated substitution in SINE elements, roughly equal rates in DNA and LTR elements, and the lowest substitution rate in LINEs. Furthermore, at non-CpG-prone sites, all simulated TE families appeared to be evolving at approximately equal rates.

This contrast would suggest that other forces (e.g., biased gene conversion or selection) may influence evolutionary rate in TEs. However, we note that these simulations are not a rigorous means of eliminating mutational variation as the cause of substitutional variation between TE families. In particular, the implicit assumption that different members of different element families were inserted into the genome simultaneously may be unrealistic.

### Divergence and Constraint

**Figure 4:**
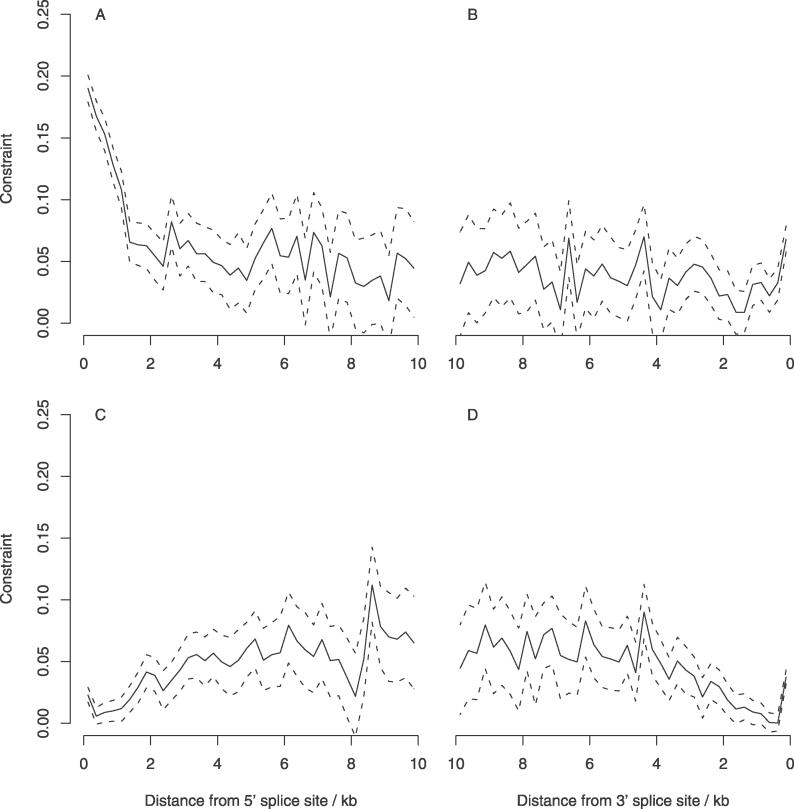
Change in Intronic Constraint with Distance from the Splice Sites Constraint was estimated at non-CpG-prone sites in first (A and B) and non-first (C and D) introns. Dashed lines show 95% confidence intervals estimated by bootstrapping the dataset by 1-Mb block, 1,000 times.

Variation in constraint and divergence with distance from splice sites in first introns is shown in Figures 4A and 4B and S3. Constraint is significantly above zero in first introns for at least the first 10 kb upstream and downstream of the acceptor and donor splice sites, respectively. As demonstrated previously [18], constraint is highest at the 5′ end of intron 1, reaching a maximum value of approximately 20% immediately adjacent to the 5′ splice site. In contrast to these previous analyses, however, adoption of a new neutral standard and a larger dataset reveals that the 3′ end of intron 1 is also under low, but significant, constraint.

In our previous analyses [18], it was assumed that the fastest evolving intron sites were neutral. This assumption, by definition, precluded the estimation of selective constraint in those introns in which the neutral standard was located. Use of ancestral repeats in the current study allowed an investigation of patterns of selective constraint within non-first introns (Figure 4C and 4D). The results show that, whilst intronic sequence situated in the first 1–2 kilobases (kb) of non-first introns is evolving at a similar rate to intronic TEs, sequence more distant than this from the splice sites is under low to moderate selective constraint. Although this may seem counterintuitive, this results from the relationship between intron length, ordinal number, and constraint (see below). Small introns (<3 kb) are the least selectively constrained and these contribute only to the estimates of divergence near to the splice sites. In contrast, longer introns, which are more highly selectively constrained, contribute to estimates of constraint both proximal and distal to intron splice sites.

**Figure 5:**
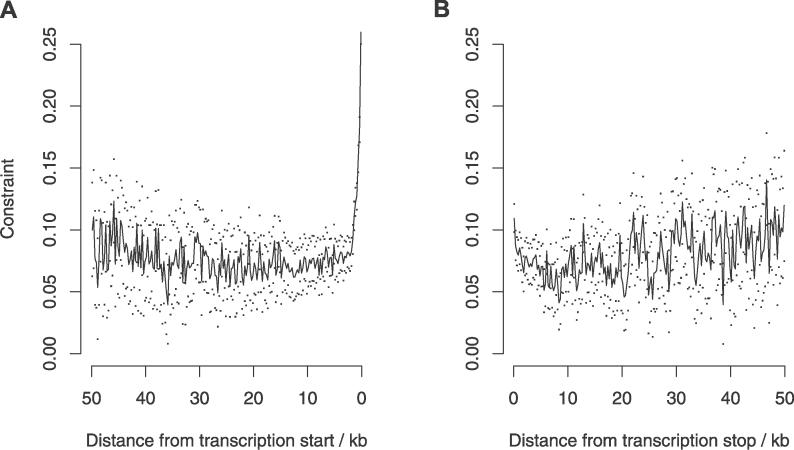
Change in Intergenic Constraint with Distance from Transcription Start and Stop Points Constraint was estimated at non-CpG-prone sites. Dots show 95% confidence intervals estimated by bootstrapping the data by 1-Mb block, 1,000 times.

The change in mean constraint (Figure 5) and pair-wise divergence (Figure S4) in intergenic DNA with distance from the transcription start/stop points was also estimated. Although there is a sharp drop in constraint immediately adjacent to the start/end of the UTRs, this appears to plateau at ~5 kb. Further into the intergenic region, constraint apparently does not drop to zero but appears to increase slightly. Although the number of sites also decreases with distance, it seems that even comparatively large distances from genic regions, alignable, nonrepetitive sites are still under moderate selective constraint. It is notable that the 95% confidence intervals of constraint estimates do not span zero at any point over a distance of 50 kb (Figure 5).

In order to calculate the relative contribution of each different sequence class to the total numbers of constrained sites in the genome, the mean selective constraint across all sites was estimated for each class (Table 1). The number of constrained bases per locus was defined as the product of the mean constraint at non-CpG-prone sites and the mean number of aligned sites per locus for that class. This method assumes that constraint at non-CpG-prone sites is a reliable estimate of constraint at all sites. To get the total number of sites, this figure was multiplied by an estimate of the total number of mouse genes. The estimate of the number of mouse genes (26,512) was based on the total number of known and predicted genes in release 36 of the ENSEMBL database [24]. A few striking patterns are evident. Firstly, whilst the estimated number of constrained, non-degenerate coding sites is not insubstantial (25 Mb), there are over three times as many constrained sites in noncoding regions (83 Mb). In addition, of all classes of noncoding DNA, the majority (~47 Mb) of constrained sites are located within the “deep” (>5 kb from known coding sequence) intergenic regions. The contribution of intronic sequence to the total number of constrained bases is, by comparison, small. Our results also show that mean intronic constraint is primarily related to intron ordinal number (see below). Finally, we find that 3′ UTR sequences contain over three times as many constrained bases as 5′ UTRs, a conclusion supported by a recent analysis of conserved elements in vertebrates [12].

**Table 1:**
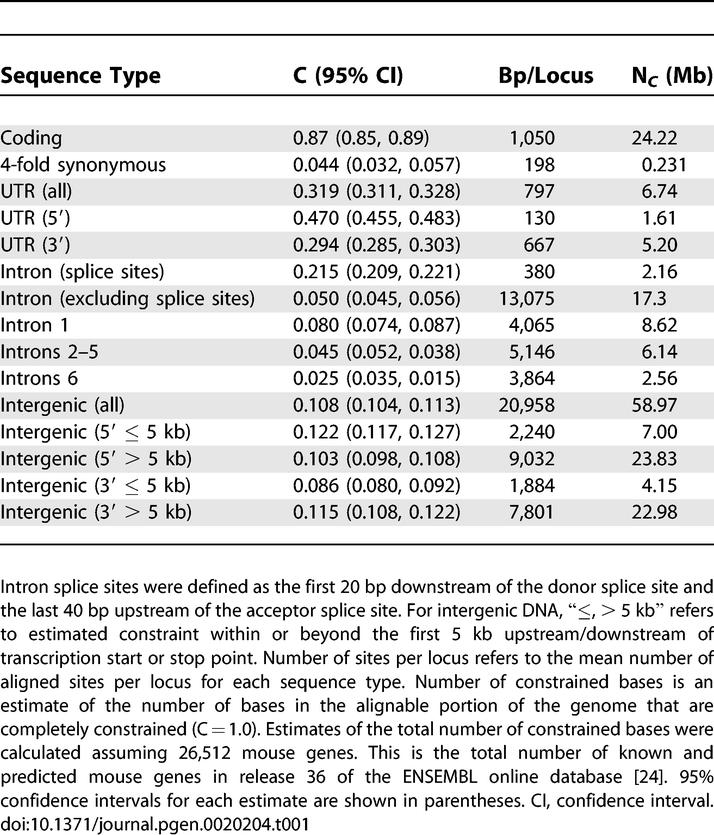
Selective Constraint and Numbers of Constrained Sites (N***_C_***) in the Murid Genome

### Intron Length, Ordinal Number, and Selective Constraint

**Figure 6:**
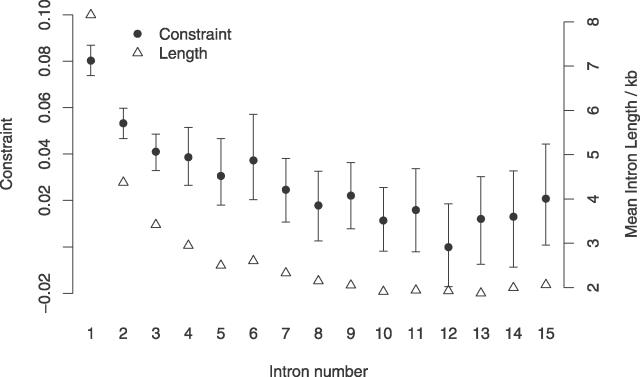
Mouse Intron Length Selective Constraint and Ordinal Number Bars show the 95% confidence interval of constraint obtained by bootstrapping the data by 1-Mb block, 1,000 times.

The results presented in Figure 4 appear to suggest a relationship between mean intron length and selective constraint. In *Arabidopsis* it has been shown that intron length is also negatively related to intron ordinal number [25]. We therefore investigated whether a similar relationship exists in murids, and how intron ordinal number relates to selective constraint. The results of this analysis are presented in Figure 6. Both mean intronic length and selective constraint are negatively associated with intron ordinal number in murids. This conclusion is supported by a recent study showing that the number of conserved blocks between human and mouse is also related to intron number [26]. Despite this, the slope of the regression line of mean intronic constraint (averaged across all introns in a gene) on gene total intron number, whilst significant, is extremely small (~3.4 × 10^−4^). This results from two factors. Firstly, because of the negative relationship between intron ordinal number and length, higher number introns have progressively smaller impact on the total intronic constraint. Thus, mean constraint in genes with large numbers of introns is primarily determined by their low number introns. Secondly, the variation in constraint within each intron number class across genes with different numbers of introns is small. For example, divergence in the first intron is not significantly different between genes with one intron and genes with 15 introns (*t*-test; *p* ≤ 0.39). This result indicates that there is a general trend across all murid genes toward the accumulation of functional intronic DNA toward the 5′ end of the gene.

### Noncoding Constraint and Gene Function

We investigated the relationship between mean intronic and intergenic constraint and gene function by classifying the genes in our dataset according to their annotated “biological process” in the PANTHER protein family database [27]. The results of this analysis are presented in Tables 2 and 3. Of our 6,381 mouse-rat gene orthologs, 3,551 could be assigned to a particular protein family or subfamily. Of these, 1,834 were assigned to multiple families or subfamilies. As so many genes in our dataset were classified as having multiple functions, ontology groups were not independent of one another and it was therefore not possible to assess (using a one-way ANOVA, for example) whether gene function has a statistically significant effect upon noncoding constraint. Nonetheless, mean intronic and intergenic constraint does appear to vary nonrandomly with gene functional group. Interestingly, our analysis supports the view that genes with complex expression patterns require proportionally more regulatory DNA. In particular, we note that genes involved in development and neuronal processes are among those associated with the highest number of putatively functional intronic and intergenic sites. In contrast, genes involved in a variety of metabolic functions contain and are surrounded by substantially less constrained noncoding DNA. The relationship between total number of putative functional intronic sites and gene ontology does not appear to be explained by differences in the median number of introns between ontology groups. This is likely to be because of the weak relationship between total intron number and constraint, as mentioned above. In addition there seems to be substantial variation within ontology groups in median intron number.

**Table 2:**
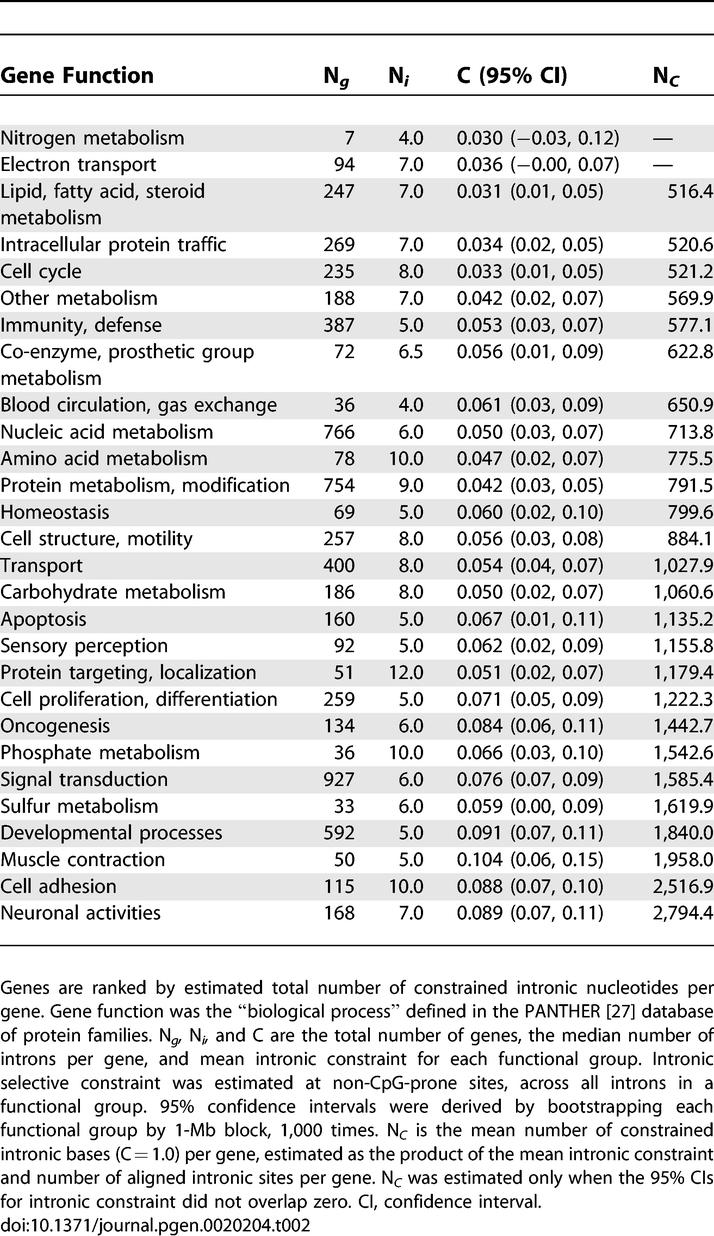
Mean Intron Number, Constraint, and Number of Constrained Intronic Bases by Gene Function

**Table 3:**
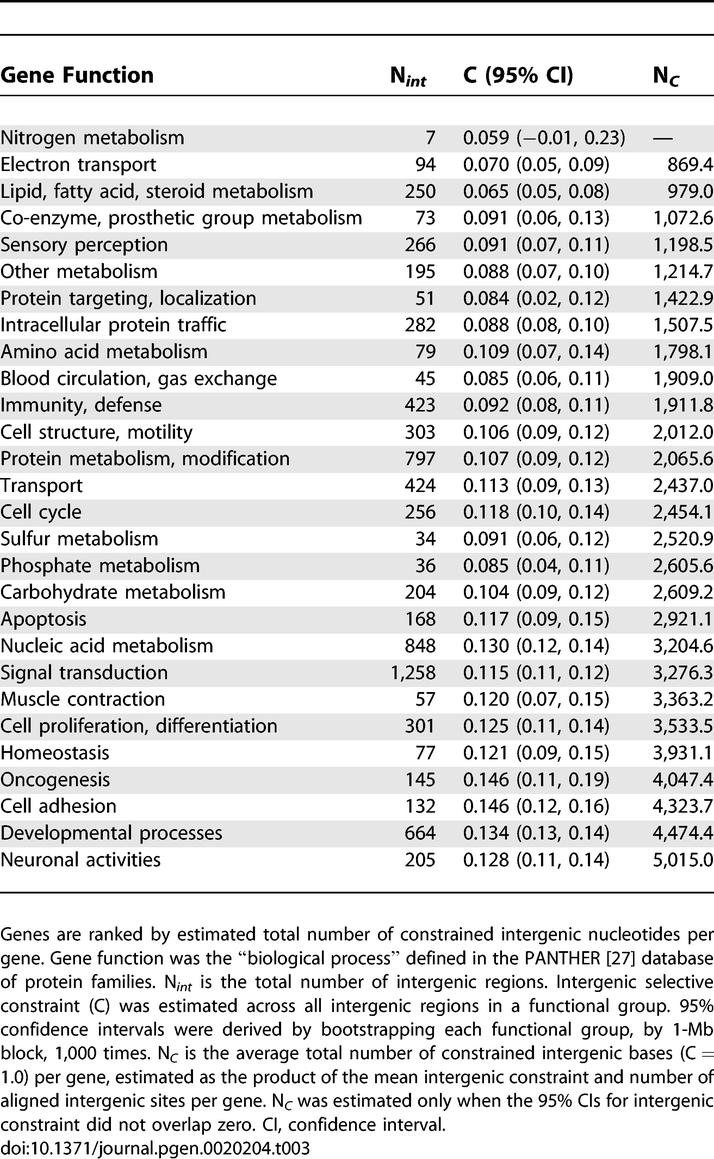
Intergenic Constraint and Number of Constrained Intergenic Bases by Gene Function

### Genomic Deleterious Mutation Rate

Our estimate of 108.48-Mb total constrained sequence (Table 1) gives an average selective constraint of 0.087 in the mouse-rat genome (see Materials and Methods). This estimate of selective constraint applies strictly to that nonrepetitive portion of the mouse and rat genomes that can be aligned with each other. By definition, we assumed that all mutations occurring within repetitive DNA are neutral. Furthermore, extension of our results to the entire mouse or rat genome requires some assumptions regarding the selective neutrality, or otherwise, of that proportion of either species' genome which is lineage- specific. Although selective constraint of lineage-specific sequence is impossible to measure using the method employed in this study, we can define a lower bound for the average constraint of a single nucleotide in the entire genome by assuming that all lineage-specific sequence in mouse is completely neutral. The estimated average selective constraint of a single nucleotide then becomes 108.48 Mb/1,462 Mb = 0.074, where 1,462 Mb is the total nonrepetitive DNA in the mouse genome.

Assuming lineage-specific sequence is under the same mean selective constraint as sequence which can be aligned between mouse and rat, we calculate that, on average, the mouse genome has experienced 0.91 deleterious mutations per generation, since divergence from the rat (Table 4). Our results would also indicate that nearly twice as many of these mutations have occurred in the nongenic portion of the genome as the genic region. This estimate of *U* is over twice as large as that estimated in a previous study (0.44) [18], despite a substantially lower estimate of the total number of mouse genes used in this study (25,612 versus 35,000). This increase is primarily due to the inclusion of larger amounts of intergenic sequence, and the exclusion of repetitive DNA from estimates of constraint. The lower bound for *U* (0.79; assuming lineage-specific sequence is entirely neutral) is also considerably larger than our earlier estimate. We note, however, that this calculation relies on parameters, such as mouse-rat divergence time, total number of mouse genes, and number of generations per year, which are themselves the subject of debate.

**Table 4:**
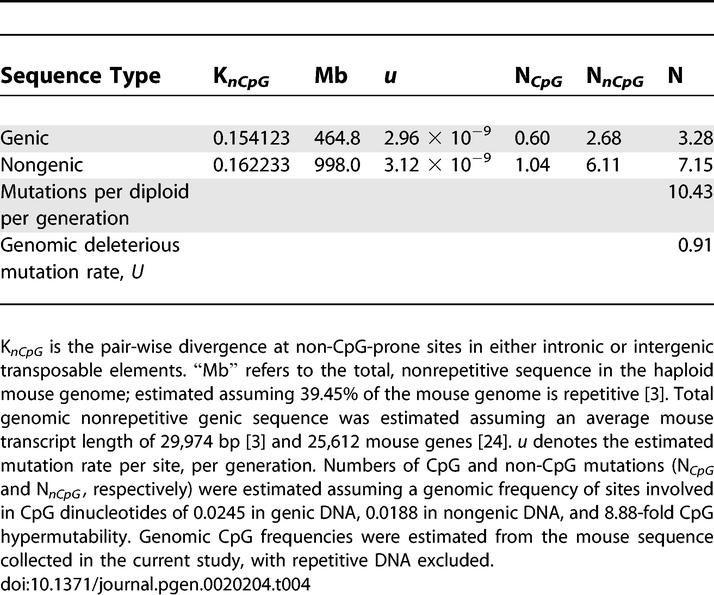
Murid Diploid Genomic Deleterious Mutation Rate

## Discussion

This study investigated genomic patterns of selective constraint in murid noncoding DNA. Our results provide estimates of genomic selective constraint in a variety of classes of murid noncoding DNA and can be summarized as follows: Firstly, the data indicate that there are at least three times as many selectively constrained, functional noncoding sites as coding sites in murids. This estimate is considerably larger than from our previous study in murids [18], but is in a similar range to that more recently estimated in humans [10] and in a variety of vertebrates [12]. Our results also indicate that intergenic selective constraint does not appear to decrease with distance from known genic regions beyond a certain distance (~5 kb). Secondly, this study clarifies the patterns and extent of evolutionary constraints within murid introns. Our results show that intronic constraint and intron length both covary with intron ordinal number. Perhaps surprisingly, however, our data suggest that selective constraint in all introns, even intron 1, is significantly lower than in intergenic regions. Thirdly, we show that the number of selectively constrained, putatively functional intronic and intergenic sites vary markedly with gene function. Interestingly, our results indicate that there are many more selectively constrained noncoding sites within and adjacent to developmental and neuronal genes than genes involved in a variety of metabolic functions. Fourthly, assuming that nucleotide substitution rates within TEs primarily reflect mutational rather than selective forces, our results suggest that mutation rates within genic DNA are lower than those in nongenic regions. Finally, taken together with our estimates of selective constraint, it is likely that over twice as many deleterious mutations have occurred in nongenic, as opposed to genic, sequence over the murid evolutionary tree.

Our study provides an insight into the evolutionary significance of introns. The results presented here suggest that there has been a general accumulation of functional intronic sites toward the 5′ end of murid genes. This pattern is apparent both within intron 1 and across all introns, with mean constraint decreasing with increasing intron ordinal number. Accumulation of functional intronic DNA is also reflected in intron length, which is negatively correlated with intron ordinal number. Previous studies in humans have shown that multi-species conserved sequences are preferentially located within longer introns [28] and this is likely to be the same phenomenon we observe in this study. Furthermore, our results suggest that the total number of functional intronic sites covaries with gene ontology, with the highest numbers of functional sites located in (among others) genes involved in development and nervous system functioning. What is the significance of these results?

Evolutionarily conserved regions within introns are likely to play (at least) two separate roles: gene splicing and gene expression control. We suggest that it is likely that most functional intronic sites in intron 1 are involved in transcription control, given their proximity to the gene promoter. Furthermore, assuming it is equally important that high and low number introns are spliced correctly, it seems improbable that intronic splice control regions should be preferentially located in lower ordinal number introns. Additionally, we have (imperfectly) excluded many alternatively spliced genes from our dataset, and so those intronic sites which regulate alternative splicing should have been excluded. Our data therefore suggest that many of the selectively constrained intronic nucleotides we observe are involved in expression control and that these sites tend to cluster toward the 5′ end of the gene in which they reside. It is also likely that many of the constrained intergenic nucleotides we observed function in the regulation of the gene they are adjacent to. Thus, our results suggest that genes involved in development, cell adhesion, and neuronal activities require more complex regulation than genes involved in electron transport, cell cycle, lipid and steroid metabolism, and a variety of metabolic processes. It may be that, in part, this pattern is determined by variation in the number of transcription factors required to control a gene's expression. For example, transcriptional control of developmental genes is finely tuned and may require rapid alternate stimulation and suppression by multiple different transcription factors. Each of these factors, in turn, binds to an individual site in the gene promoter region, and so genes which are regulated by large numbers of transcription factors may be associated with larger numbers of constrained noncoding sites.

This conclusion is supported by evidence suggesting that the quantity of noncoding DNA in Caenorhabditis elegans and Drosophila melanogaster genes is related to their regulatory complexity [29]. One potential implication of this result is that there will be a substantially higher deleterious mutation rate in the regulatory noncoding DNA of genes with complex regulation than in those which are permanently “switched on.” For example, summing across intergenic and intronic DNA, we estimate that a developmental or neuronal gene is associated with, on average, almost six times as many constrained functional sites as a gene involved in electron transport or lipid or steroid metabolism. If most of these sites are involved in the regulation of the adjacent gene, and assuming base mutation rates are similar across different ontology groups, then developmental and neuronal genes may be up to six times more likely to experience a deleterious regulatory mutation than genes involved in electron transport or lipid metabolism. It is clear, however, that a deeper understanding of the nature of the motifs that regulate gene expression and control mRNA splicing will shed light on the relative significance of these two functions in explaining patterns of intronic constraint.

Our study adds to the growing body of work that indicates that mammalian synonymous sites are under some purifying selection [30–32], although it seems that, in murids at least, mean selective constraint at such sites is extremely weak. This may be an underestimate, however, if, as suggested by our simulations, 4-fold sites have a higher non-CpG mutation rate than other regions of the genome. The GC content at 4-fold sites is elevated beyond that typical in noncoding DNA (~53%) and this may reflect codon bias towards G- or C-ending codons, as has been reported in humans [33]. Constraint at 4-fold sites could also result from purifying selection on exonic splice enhancers [34].

The accuracy of our estimates of constraint relies on the assumption that TEs can be used to estimate the local, neutral mutation rate. Our simulations appear to suggest that the variation we observe in mean nucleotide substitution rate between TE families reflects non-neutral evolution. However, there are a number of scenarios, both neutral and otherwise, which could explain this observation. Our simulations may have neglected some important aspects of TE evolution. For example, it may be that different TE families insert into regions with different mean mutation rates. It is known that SINE elements are preferentially located within GC rich regions in the human genome, whereas LINE elements tend to occur in AT rich regions [1]. If these varying base compositions are the result of regional mutational variation, then this could explain the differences in substitution rates between TE families. In this case the assumption that TEs can be used to estimate local mutation rates holds.

There are also a number of scenarios which explain evolutionary rate variation between TE families in which the assumption that TEs reliably reflect local mutation rates is violated. TE evolutionary rate may covary with age of insertion, as older TEs evolve more slowly after coming to compositional equilibrium with their surroundings [33]. If this is the case, we may have overestimated constraint by including relatively recently inserted TEs in our dataset. In addition, TEs may occasionally acquire a selectively beneficial function [35–37] and thus be preserved by purifying selection. Finally, it may be that substitution rates in TEs are influenced by biased gene conversion [38] such that mutations increasing GC content are fixed preferentially. The effect of biased gene conversion is equivalent to selection for the allele toward which gene conversion is biased [39]. If substantial selection or biased gene conversion is occurring, it is likely that we have underestimated constraint. However, although there have been a number of recent discoveries of selectively co-opted TEs, these functional TEs still represent a small minority of most extant TEs. Furthermore, in this study it appears that repetitive DNA is the most swiftly evolving sequence class. Thus, if such processes regularly occur in TEs their effects are either small, confined to a minority of elements within the dataset, or both.

A further caveat to our estimates of genomic constraint relates to the exclusion of potentially alternatively spliced genes from the dataset. It is known that introns within alternatively spliced genes are more highly conserved than in constitutively spliced genes [40]. If such conserved regions are truly noncoding, as opposed to being occasionally included in an alternate exon, then our estimates of intronic constraint may also be biased downward. We note also that our extrapolation of estimates of constraint to the whole murid genome will potentially be biased upward by two factors. Firstly, we have used sequence similarity (via BLAST comparison) as a criterion of orthology and this will necessarily bias our dataset toward more evolutionarily conserved genes. However, until a relatively complete functional annotation of the mouse and rat genomes becomes available, this criterion remains necessary. Given the low threshold E-value we used and the relatively small divergence between mice and rats, we argue that this should have a relatively small impact on our results. Of perhaps more concern is the presence of overlapping genes in our dataset. Overlapping genes will share some common intergenic and intronic sequence, which means that our method of extrapolation to the whole genome will overestimate the quantity of constrained noncoding sequence. Some recent studies have suggested that antisense transcription is a common phenomenon in the human genome [41]. It is difficult to assess the impact of overlapping genes on our data, but it could be substantial. Finally, we will have underestimated the genomic deleterious mutation rate since we have not considered indel mutations, particularly transposable element insertions, at least some of which are known to have deleterious effects [42,43]. Whilst the effects of indel mutations in coding sequence are probably unconditionally deleterious, the impact of indel mutations in noncoding DNA is still poorly understood.

## Materials and Methods

### Data collection.

A list of “known” mouse peptide sequences was obtained from release 36 of the ENSEMBL sequence database [24]. This list consists of peptide models that can be mapped to mouse-specific peptides in the Swiss-Prot, RefSeq, or SPTrEMBL databases. In this study we used only those peptides that matched an existing sequence in the National Center for Biotechnology Information (NCBI) RefSeq database. We removed those peptides which were annotated in ENSEMBL as having multiple transcripts, due to the uncertainty of annotation of introns in alternatively spliced genes. Finally, those peptides which were not listed as having a unique best reciprocal BLAST hit (UBRH) in the rat genome in the ENSEMBL database were removed. The remaining peptides were then mapped onto NCBI build 33.1 of the mouse genome using their RefSeq IDs, and their coding sequences extracted.

Putative rat orthologs of the mouse coding sequences were located by comparing the first and last exons of each mouse coding sequence to NCBI build 3.1 of the rat genome using BLASTN [44]. Mouse coding sequences were only compared with regions of the rat genome that are known to be syntenic, where synteny was as defined in Figure 4 of [3]. The “flanking” sequences extended to the midpoint in intergenic DNA to the next annotated mouse coding sequence. BLAST matches in the rat genome were accepted or rejected on the basis of a number of criteria. Firstly, coding sequences were excluded unless both first and last exons had a single unique match on the same rat contig. Secondly, if both first and last mouse exons matched more than one sequence on multiple, different rat contigs they were excluded. Thirdly, both first and last exon matches were rejected unless BLAST hits were matched on the same strand of the rat genome. Fourthly, matches of the first and last mouse exons, which were further than 1 Mb apart on the same rat contig, were also rejected. Finally, only matches for which the BLAST E-value of both first and last exons was ≤ 10^−10^ were accepted. Whilst these reduced the number of genes in our dataset, we argue that they are necessary limitations of our analysis.

For each pair of flanking mouse sequences and their corresponding matches in the rat, we extracted all sequences between the start of the upstream flank to the end of the downstream flank. This pair of rat and mouse sequences was aligned using AVID (45). We located LINEs, SINEs, LTRs, and DNA insertion elements in these alignments using RepeatMasker (http://www.repeatmasker.org). Mouse repeats which were well aligned with the corresponding region in the rat sequence were denoted as being ancestral or inserted prior to the mouse-rat divergence. In this context, “well-aligned” was defined as those mouse-rat repeat alignments which contained more than 30 valid (i.e., not masked; see below), aligned nucleotides. This criterion was deliberately lax in order to avoid potential selection bias toward more conserved sequences. The efficacy of this criterion in identifying reasonably aligned repeats was confirmed by eye for a subset of the total dataset. In our final dataset, less than 5% of the total number of ancestral repeat alignments contained less than 55 valid, aligned nucleotides.

Aligned coding and intronic sequence was also extracted, using the annotated mouse exons as a reference. Any mouse or rat coding sequence that did not have a valid start and stop codon, or included premature stop codons was excluded. The remaining coding sequences were realigned using CLUSTALW [46], an alignment method that was specifically designed for the alignment of protein-coding genes.

### Alignment masking.

In order to minimize the possibility of nonorthologous sites contributing to estimates of divergence, a simple masking protocol was implemented. Two primary masking targets were identified: (i) Sections of alignments which were so divergent as to be unlikely to be orthologous were located through the use of a sliding window of 40 bp in size. Any region in which each of 30 or more consecutive windows showed a mean divergence greater than the threshold divergence of 30% was masked. The divergence threshold was set to be three standard deviations above the mean divergence of ancestral repeats (mean = 0.1596; standard deviation = 0.0504). (ii) Regions which contained short aligned blocks surrounded by large gaps were also considered unlikely to be truly nonhomologous and were masked off. These regions were identified as one or more blocks of <20 bp in size, flanked by large gaps (>40 bp) in size. Any alignments which contained ≥75% putatively nonorthologous sites as identified by these criteria were excluded from further analyses. In addition to masking putatively nonorthologous sites, repetitive sequence (simple sequence repeats, retroelements, and DNA elements) present in the alignments were also masked using RepeatMasker, as this study specifically addresses constraint within unique, nonrepetitive sequence.

### Data analysis.

Nucleotide substitution rates were corrected for multiple hits according to the Tamura-Nei model [47]. It has been suggested that the level of methylation may differ between repetitive and nonrepetitive DNA [48]. If this is the case, CpG sites effectively mutate at different rates depending upon their location in the genome and it is desirable to remove this effect as much as possible from the estimation of selective constraint. In order to effectively remove the impact of CpG-derived mutation, nucleotide substitution rates were also calculated at those sites that were either preceded or followed by a “C” or a “G” (non-CpG-prone sites). Substitution rates at linked sites are also autocorrelated across distances of ~1 Mb in murids [15]. All gene orthologs were therefore grouped into 1-Mb blocks, according to their annotation on the mouse genome, to minimize the effects of autocorrelation of substitution rates on the estimation of standard errors and confidence intervals. These 1-Mb blocks were treated as independent observations in the dataset. Substitution rates in different sequence types were estimated by summing across all annotated regions of interest (e.g., all non-first introns or intergenic transposable elements) within a block. Synonymous substitution rates were estimated at 4-fold degenerate sites only. Only those 4-fold codons that coded for the same amino acid in both derived sequences and that had experienced a single synonymous change were defined as ancestrally 4-fold. All standard errors and confidence intervals were calculated by bootstrapping the data over a 1-Mb block, 1,000 times.

In order to estimate selective constraint, a variation of the method of Kondrashov and Crow [49] was employed, as in previous studies [17,18,50]. For each sequence class, observed substitution rates were compared to that expected under neutrality, where the neutral expectation was estimated using a weighted average of the substitution rate in all ancestral repeats within a block. However, when base composition varies between assumed neutrally evolving sequence and the sequence of interest, differences in the frequencies of each nucleotide can introduce error into the estimation of the expected evolutionary rate under neutrality. We attempted to account for this by estimating the expected substitution rates for different nucleotides separately. The Tamura-Nei model is described by three substitution rate parameters: the A↔G transition rate *(K_AG_),* the T↔C transition rate *(K_TC_),* and the transversion substitution rate *(K_TV_).* The rate of substitution expected under neutrality was calculated as the product of each of the mean ancestral repeat substitution rates and the number of appropriate bases for that substitution type in the target sequence of interest. We have:

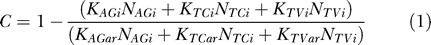
where K. i denotes the substitution rate in the sequence of interest, N. i the number of sites of a certain type in the sequence of interest, and K. ar the mean substitution rate (weighted by length) across all ancestral repeats located within a block. In all cases, constraint was estimated at non-CpG-prone sites, both to remove the influence of differential CpG frequency between sequence classes as well as to avoid the potential effects of differential methylation of repetitive and nonrepetitive DNA. When investigating the relationship between sequence length and selective constraint, sequence length was always defined as the number of bases in mouse.

### Simulations.

**Figure 7:**
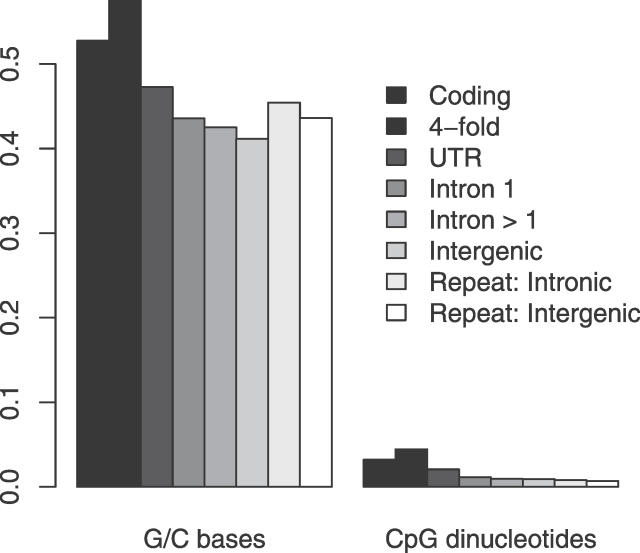
Proportion of G/C Bases and CpG Dinucleotides in Different Mouse Sequence Classes

We attempted to quantify the effects of compositional variation on rates of substitution using a simulation approach. Sequences were divided into three approximate groups on the basis of their observed GC and CpG contents: 4-fold degenerate, intronic/intergenic, and ancestral repeat (Figure 7). In order to accurately reflect the base composition of coding and noncoding sequence, simulated phylogenies were generated using real mouse sequence data. All mouse coding sequences and a random sample of mouse intronic and repetitive sequences (of approximately the same length as that of the coding DNA used, i.e., ~8.5 Mb) were concatenated into a single sequence. This sequence was evolved along two independent branches to produce a tree in which the probability of a nucleotide substitution at any site in either lineage was 0.08, on average. 80% of amino acid changing mutations in coding sequence were rejected. The remaining sites (4-fold degenerate, intronic, and repetitive) were allowed to evolve neutrally. A different random sample of intronic and repetitive sequence was collected for each replicate phylogeny.

The mutation model implemented in all our simulations was derived from mouse polymorphism data extracted from the NCBI single nucleotide polymorphism (SNP) database (http://www.ncbi.nlm.nih.gov/projects/SNP). We collected all non-redundant refSNP clusters for each mouse chromosome, excluding those SNPs which were inferred from sample sizes of less than 20 individuals. The flanking sequence of each SNP was then compared to a BLAST database constructed from all mouse non-first introns of length ≤6 kb on the appropriate mouse chromosome. We used these introns as they are likely to be evolving close to neutrally (see Table 1). Only those SNPs whose flanking sequence returned a single, significant (e ≤ 10^−50^; ≥95% sequence identity) match to a sequence in the intron BLAST database were included in our final dataset. In order to estimate the mutation frequencies, we assumed that the most frequent allele was ancestral.

Using these data, the relative mutabilities at three site types (CpG, non-CpG “G”, or “C” and non-CpG “A”or “T”) were estimated, by pooling the total number of polymorphisms inferred to have occurred at one of three site types. The relative mutabilities of the three site types were estimated as 0.815, 0.100, and 0.085, respectively. The relative probabilities, given that a mutation occurs, of six different non-CpG mutation types and three CpG mutation types, were also derived. The six non-CpG mutation types inferred from polymorphism data were transversions within Watson-Crick pairs (A↔T, G↔C), transversions between Watson-Crick pairs (A↔C or T↔G, C↔A or G↔T), and transitions between Watson-Crick pairs (G↔A or C↔T, A↔G or T↔C). Three CpG mutation types were also inferred from the polymorphism data. These mutational classes were chosen because it is impossible to infer from sequence data which strand a mutation originally occurred upon. The relative probabilities used in the simulation are presented in Table S1. Assuming that this mutation model accurately represents true murid point mutation rates, simulated phylogenies should approximate the rates and patterns of nucleotide substitution expected under neutral evolution of 4-fold sites, introns, and transposable elements.

Preliminary results suggested that the nucleotide substitution rate varies between different transposable element families. In order to determine whether this variation could be the result of compositional variation coupled with mutational bias, we simulated the evolution of the four main transposable element families: SINEs, LINEs, LTRs, and DNA transposons. In these simulations we used transposable element consensus sequences from the rodent RepeatMasker Libraries (http://www.girinst.org) as the ancestral sequence. In each simulated replicate, a single consensus sequence was chosen at random for each of the four transposable element families. These four sequences were concatenated into a single sequence which was evolved neutrally along two lineages as described above.

### Noncoding constraint and gene ontology.

Genes with different functional roles and expression levels appear to harbor (and be surrounded by) different quantities of noncoding DNA [51,52]. There are at least two (non-mutually exclusive) factors which may produce this variation. Firstly, intron length (and the length of all noncoding DNA) may be dictated simply by the amount of functional noncoding sequence which it contains [26,53]. Secondly, an intron's length may be affected by selection for transcriptional efficiency, and therefore be determined by the expression level of the gene in which it lies [25,51]. It is likely that both these forces play a role in shaping the length of noncoding DNA. Of relevance to this issue is how intronic constraint varies (if at all) with gene function. In particular, given that our analysis revealed a relationship between intron length, ordinal number, and constraint, we wanted to determine how these variables relate to the functional role of a gene.

To this end, we classified the genes in our dataset according to function based on their biological classification (i.e., “biological process”) in the PANTHER protein family database [27]. We used the RefSeq IDs of the genes in our dataset to determine their function as annotated in the PANTHER database. We note that a proportion of the genes in our dataset are annotated as having multiple functions.

### Genomic deleterious mutation rate.

The estimation of genomic selective constraint enables us to calculate the diploid genomic deleterious mutation rate per generation *(U),* an important parameter in population genetics. We assume that mouse and rat diverged 13 million years ago [54], and that mice, on average, undergo two generations per year [50]. In order to estimate the average constraint upon a single nucleotide, we assume a total of 1,241-Mb nonrepetitive sequences can be aligned between mouse and rat [3]. When calculating the genomic mutation rate per genome per generation, we accounted for CpG hypermutability by calculating the contributions from these sites separately. From the mouse polymorphism data we estimated that CpG-derived mutations occur at 8.88 times the rate of non-CpG mutations. We further divide the contribution of mutations occurring in genic and nongenic regions, given that our data suggest that the non-CpG mutation rate differs between both regions. For both, we use the mean transposable element pair-wise divergence at non-CpG-prone sites to estimate the non- CpG mutation rate per site.

## Supporting Information

Figure S1 Constraint in Simulated Neutrally Evolved Sequence Distribution of “constraint” values estimated in neutrally evolved, simulated noncoding DNA, using transposable elements evolved under the same mutational model as a “neutral standard.” Constraint was estimated for each one of 100 replicates.
(3 KB EPS)

Figure S2 Substitution Rates in Simulated Transposable Elements Estimated mean nucleotide substitution rate in simulated SINEs, LINEs, LTRs, and DNA elements. Estimates are averaged across 10,000 simulated replicates.
(3 KB EPS)

Figure S3 Pair-Wise Divergence in Introns Pair-wise divergence at non-CpG-prone sites in 50-bp blocks in intron 1 (A) and non-first introns (B), upstream and downstream of 3′ and 5′ splice regions. Dots show the 95% confidence intervals for each block substitution rate and were estimated by bootstrapping the data by 1Mb block, 1,000 times. The dashed line shows the mean divergence of intronic transposable elements.
(62 KB EPS)

Figure S4 Pair-Wise Divergence in Intergenic DNA Pair-wise divergence at non-CpG-prone sites in 50-bp blocks in intergenic DNA upstream (A) and downstream (B) of annotated transcription start points. Dots show the 95% confidence intervals for each block substitution rate and were estimated by bootstrapping the data by 1Mb block, 1,000 times. The dotted line shows the mean divergence of intergenic transposable elements.
(144 KB EPS)

Table S1. Simulation Mutation Matrix Click here for additional data file. Mutation matrix implemented in the simulations. Each element *ij* in the matrix gives the relative probability of nucleotide in row *i* mutating to the nucleotide in column *j.* Italicized figures denote the relative probability of the given mutation occurring at one of the sites within a CpG dinucleotide.
(29 KB DOC)

## Abbreviations

kb: kilobase
LINE: long INterspersed element
LTR: long terminal repeat transposon
Mb: megabase
SINE: short INterspersed element
TE: transposable element
