# Supplementary material for: Genomic Selective Constraints in Murid Noncoding DNA
Source: PLoS Genet. 2006 Nov 24;2(11):e204. doi: 10.1371/journal.pgen.0020204 (PMC1657059; doi:10.1371/journal.pgen.0020204)
Supplement: Table S1. Simulation Mutation Matrix [file pgen.0020204.st001.doc]

**Table S1.**

Table S1: Simulation mutation matrix.

|  | A | T | G | C |
| --- | --- | --- | --- | --- |
| A | - | 0.146 | 0.689 | 0.166 |
| T | 0.146 | - | 0.166 | 0.689 |
| G | 0.691 *(0.815)* | 0.165 *(0.106)* | - | 0.144 *(0.080)* |
| C | 0.165 *(0.106)* | 0.691 *(0.815)* | 0.144 *(0.080)* | - |

Mutation matrix implemented in the simulations. Each element *ij* in the matrix gives the relative probability of nucleotide in row *i* mutating to the nucleotide in column *j*. Italicised figures denote the relative probability of the given mutation occurring at one of the sites within a CpG dinucleotide.
